# Localized alopecia and suppression of hypothalamic-pituitary-adrenal (HPA) axis in dogs following treatment with difluprednate 0.05% ophthalmic emulsion (Durezol®)

**DOI:** 10.1186/s12917-021-03072-9

**Published:** 2021-12-01

**Authors:** Katelin Quantz, Amanda L. Anderson, Christine D. Harman, Erica L. Noland, Jacquelyn M. Del Valle, Laurence M. Occelli, Jessica B. Burn, Simon M. Petersen-Jones, Daniel K. Langlois, Chris G. Pirie, Annette D. Petersen, András M. Komáromy

**Affiliations:** 1 Department of Small Animal Clinical Sciences, Michigan State University, Veterinary Medical Center, 736 Wilson Road, East Lansing, MI 48824 USA; 2 Department of Pathobiology and Diagnostic Investigation, College of Veterinary Medicine, Michigan State University, East Lansing, MI USA; 3 Campus Animal Resources, Michigan State University, East Lansing, MI USA

**Keywords:** Alopecia, Canine, Corticosteroids, Difluprednate (Durezol®), Follicular atrophy, Hypothalamic-pituitary-adrenocortical (HPA) axis, Ophthalmic

## Abstract

**Background:**

Despite the common use of topical ophthalmic corticosteroids in dogs, detailed reports on systemic and dermatologic adverse effects are limited.

**Results:**

Nine purpose-bred research Beagles were treated with difluprednate 0.05% ophthalmic emulsion in one or both eyes 2–3 times daily. Some difluprednate treated dogs developed mild to severe alopecia of the periocular region, face, and distal pinna (5/9). The median duration of treatment prior to onset of dermatologic signs for difluprednate treated dogs was 550 days (453–1160 days). Diagnostic testing included complete blood count (CBC) and serum biochemistry, adrenocorticotropic hormone (ACTH) stimulation testing combined with endogenous ACTH measurement, and skin biopsy. The CBC and chemistry were within normal limits for all dogs. There were varying degrees of suppression of the hypothalamic-pituitary-adrenocortical (HPA) axis with difluprednate treatment. Dogs with the most profound alopecic changes had less pronounced HPA axis suppression compared to dogs with no integumentary changes. Skin biopsies demonstrated follicular atrophy and follicular keratosis. When topical difluprednate was reduced to unilateral therapy, the hair regrew on the untreated side of the face. In addition to the affected research dogs, a 7-year old female spayed Chihuahua that was being treated as a clinical patient with long-term difluprednate 0.05% ophthalmic emulsion developed generalized hypotrichosis on the head and body and a potbellied appearance. ACTH stimulation testing revealed suppression of the HPA axis with a mild increase in serum alkaline phosphatase (ALP) activity and a urine specific gravity of 1.016. The combination of clinical signs and laboratory abnormalities was supportive of iatrogenic hyperadrenocorticism.

**Conclusions:**

In dogs long-term use of difluprednate ophthalmic emulsion results in HPA axis suppression and in some cases iatrogenic hyperadrenocorticism. A novel pattern of localized alopecia is suspected to be related to dermal absorption and local action due to superior potency and penetration compared to other commonly utilized ophthalmic corticosteroids.

**Supplementary Information:**

The online version contains supplementary material available at 10.1186/s12917-021-03072-9.

## Background

Iatrogenic hyperadrenocorticism is a well-recognized complication of long-term corticosteroid administration in dogs [1, 2]. A diagnosis of iatrogenic hyperadrenocorticism is confirmed by documenting hypothalamic-pituitary-adrenocortical (HPA) axis suppression in a glucocorticoid-treated dog that has clinical or biochemical features of hyperadrenocorticism. Evidence of suppression of this axis is reflected by a decrease in endogenous adrenocorticotropic hormone (ACTH) and cortisol concentrations, and a diminished responsiveness to exogenous ACTH administration [1]. Iatrogenic hyperadrenocorticism is most common in dogs treated with high doses of oral glucocorticoids, although suppression of the HPA axis, adrenal cortical atrophy, and iatrogenic hyperadrenocorticism have been documented following treatment with ophthalmic corticosteroids [3–8]. Skin atrophy, alopecia, and comedones have also been described following use of topical glucocorticoid preparations [9]. However, localized periocular alopecia has not been reported as a sequela to use of ophthalmic corticosteroid preparations.

Difluprednate is a synthetic derivative of prednisolone and a potent glucocorticoid. The fluorination of difluprednate at the C-6 and C-9 positions results in high glucocorticoid receptor binding affinity, and the addition of a butyrate and acetate ester at the C-17 and C-21 positions results in superior tissue penetration [10–12]. Comparison between difluprednate 0.05% ophthalmic emulsion and other ophthalmic corticosteroids has been performed to evaluate its clinical efficacy in dogs utilizing a well-defined aqueous paracentesis-induced uveitis model [13, 14]. When compared to betamethasone 0.1% ophthalmic solution, difluprednate exhibited immediate superior anti-inflammatory properties [11]. A similar assessment comparing difluprednate 0.05% ophthalmic emulsion to prednisolone acetate 1% ophthalmic suspension demonstrated equivocal suppression of uveitis in a group of Beagles when followed for 5 consecutive days following paracentesis [15].

Preclinical safety testing was performed for difluprednate 0.05% ophthalmic emulsion in both rabbits and dogs, and following treatment both species developed reduced lymphocyte counts [16, 17]. This was attributed to the systemic effects of the corticosteroid [17]. The United States Food and Drug Administration (FDA) label for Durezol® reported that some animals treated with this difluprednate 0.05% ophthalmic emulsion also developed adrenal gland atrophy and thinning of the skin [18]. Our purpose was to describe the clinical and biochemical abnormalities in a group of laboratory Beagles and one client owned Chihuahua that developed varying degrees of alopecia and suppression of the HPA axis following administration of difluprednate 0.05% ophthalmic emulsion.

## Results

### Laboratory Beagles

Thirteen purpose-bred adult Beagles were included in this study, both normal controls (*n* = 4) and *ADAMTS10* mutants with open-angle glaucoma (OAG; *n* = 9) (Table 1) [19]. Of the 13 dogs, 11 were concurrently enrolled in ocular gene therapy studies involving intracameral or intravitreal, uni- or bilateral administration of adeno-associated virus (AAV) vector (Table 1). The nature of the gene therapy work, which indicated the use of long-term corticosteroids, will be described in detail in future reports. Reported corticosteroid-related findings were not affected by the gene therapy because they also occurred around corticosteroid-treated control eyes not injected with AAV.

**Table 1:** Dogs included in the study

Dog	Signalment	Weight (kg)	*ADAMTS-10* Genotype (Phenotype)	AAV	Topical Steroid Treatment	Duration of Treatment	Description of Hair Coat Abnormalities
1^a^	3 years; M Beagle	12.6	Wildtype (normal)	OS	Difluprednate OU BID	764 days	Mild periocular alopecia OU
2	2 years; M Beagle	17.6	Homozygous mutant (OAG-affected)	OU	Difluprednate OU BID	102 days	None
3^a^	5 years; M Beagle	13.6	Homozygous mutant (OAG-affected)	OS	Difluprednate OU TID	1164 days	Generalized hypotrichosis and mild periocular alopecia OU
4	5 years; M Beagle	14.3	Homozygous mutant (OAG-affected)	OU	Difluprednate OU BID	102 days	None
5	2 years; F Beagle	10.3	Wildtype (normal)	OD	Difluprednate OD BID	667 days	Periocular alopecia OD
6	4 years; F Beagle	9.6	Homozygous mutant (OAG-affected)	OD	Difluprednate OD BID	764 days	Periocular alopecia OD and pinna alopecia AU
7	4 years; F Beagle	9.75	Homozygous mutant (OAG-affected)	OS	Difluprednate OS BID	764 days	Periocular alopecia OS, pinna alopecia AS, left palmar metacarpal alopecia
8	1.5 years; F Beagle	13.85	Homozygous mutant (OAG-affected)	OD	Difluprednate OU TID	102 days	None
9	1.5 years; M Beagle	11.8	Homozygous mutant (OAG-affected)	OS	Difluprednate OU TID	102 days	None
10	4 years; MN Beagle	12.6	Homozygous mutant (OAG-affected)	OU	None	N/A	None
11	4 years; F Beagle	10.8	Homozygous mutant (OAG-affected)	OU	None	N/A	None
12	3 years; F Beagle	12.25	Carrier (normal)	–	None	N/A	None
13	3 years; M Beagle	12.6	Wildtype (normal)	–	None	N/A	None
14	7 years; FS Chihuahua	5.5	Wildtype (normal)	–	Difluprednate OU BID	413 days	Generalized hypotrichosis

*Abbreviations*: AD right ear; AS, left ear; AU, both ears; OAG, open-angle glaucoma; BID, twice daily; TID, three times daily; OD, right eye; OS, left eye; OU, both eyes; M, male; F, female; FS, female spayed; MN, male neutered

^a^ = Dogs initially treated with NPD and then subsequently transitioned to difluprednate

AAV: All dogs intracameral, except for dog 8 OD, dog 10 OU, dog 11 OU – intravitreal

All dogs receiving ophthalmic corticosteroids were treated with difluprednate 0.05% ophthalmic emulsion (Durezol®; Alcon Laboratories Inc.; Fort Worth, TX). Seven dogs were treated exclusively with difluprednate, while two dogs (Dogs 1 and 3) were initially started on neomycin-polymyxin B-dexamethasone 0.1% ophthalmic ointment (NPD; Bausch & Lomb Incorporated; Tampa, FL) for at least 1 month of therapy, and then were transitioned to difluprednate (Table 1). Treatment was administered 2–3 times daily in one or both eyes. Corticosteroid dosing frequency was adjusted to control clinical signs of gene therapy-related anterior uveitis, such as aqueous flare and cells (Table 1). Four additional dogs not receiving ophthalmic steroid treatment were included as controls for laboratory testing: two AAV-treated *ADAMTS10*-mutants (Dogs 10 and 11) and 2 normal controls (Dogs 12 and 13). Dogs 10 and 11 had previously received transient NPD treatment OU for approximately 1 month immediately after AAV-treatment and had been off all corticosteroid treatment for more than 1 year when the ACTH-stimulation testing was performed.

**Fig. 1:**
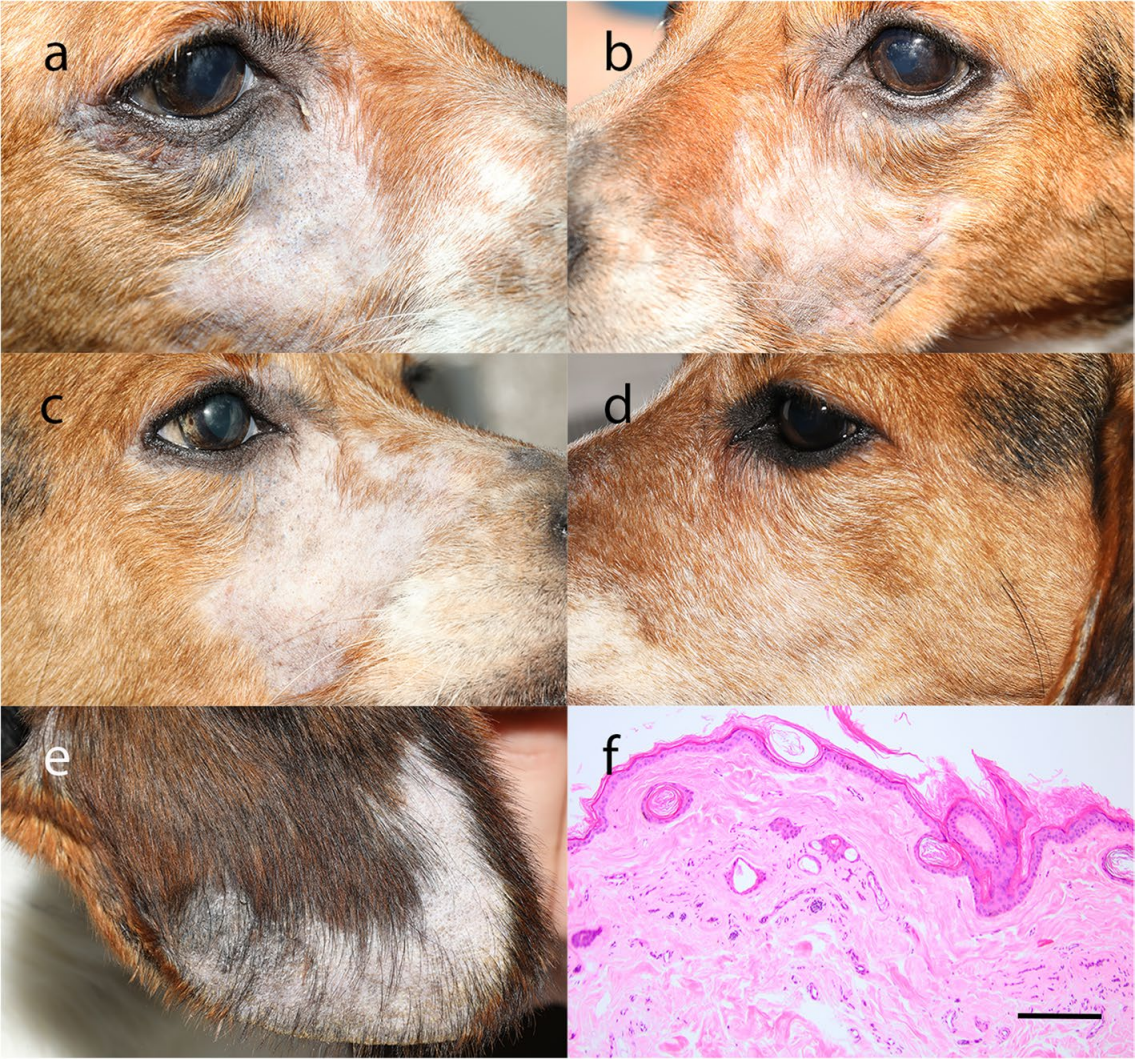
Clinical and histopathology images from Dog 6. Significant bilateral periocular and facial alopecia following treatment with difluprednate OU BID for approximately 16 months (**a**,**b**). Unilateral regrowth of hair on right side of face approximately 5 months following discontinuation of difluprednate OS (**c**,**d**). Alopecia of the distal pinna AD while receiving difluprednate OD BID (**e**). Photomicrograph from a biopsy of the left ear while receiving difluprednate OU BID (H&E) (**f**). There is severe follicular atrophy, moderate follicular keratosis, and moderate orthokeratosis. Scale bar = 400 μm

**Fig. 2:**
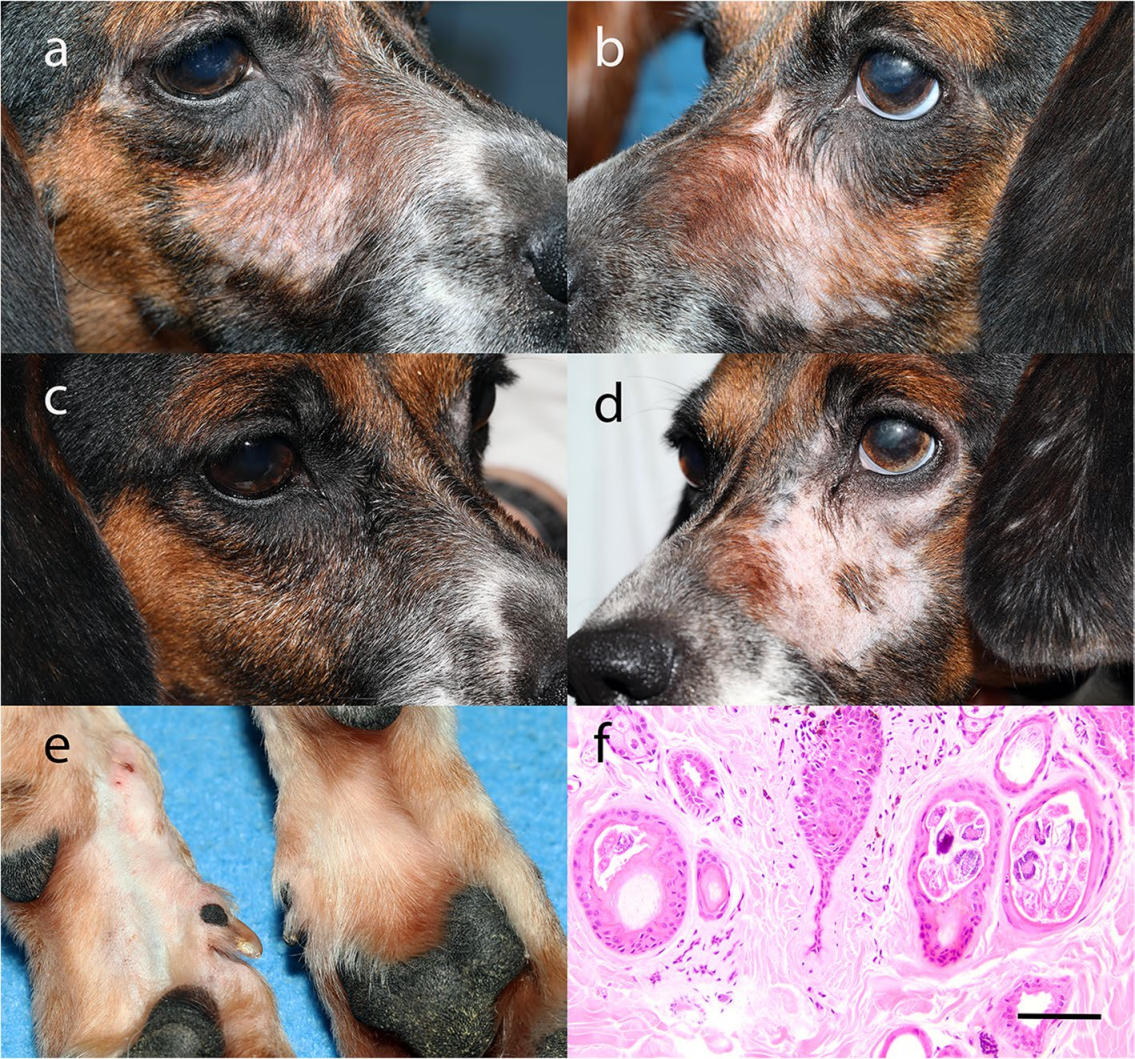
Clinical and histopathology images from Dog 7. Significant bilateral periocular and facial alopecia following treatment with difluprednate OU BID for 16 months (**a**,**b**). Unilateral regrowth of hair on right side of face approximately 5 months following discontinuation of difluprednate OD (**c**,**d**). Alopecia of the left palmar metacarpus while receiving difluprednate OS BID (**e**). Photomicrograph from a skin biopsy of the right cheek while receiving difluprednate OU BID (H&E) (**f**). There is severe follicular atrophy, mild/moderate follicular keratosis, multifocal intrafollicular mites consistent with *Demodex* and mild/moderate orthokeratosis. Scale bar = 200 μm

Periocular, facial, and less frequently pinna alopecia was observed in 5/9 dogs treated with difluprednate 0.05% ophthalmic emulsion (Table 1, Figs 1 and 2). This was initially appreciated during routine ophthalmic examination in dogs with significant dermatologic signs (Dogs 5, 6, and 7) (Figs. 1 and 2), and then subsequently in dogs with milder degrees of alopecia of the periocular region (Dogs 1, 2, and 3) (Table 1). Other, less frequently observed dermatologic abnormalities included mild generalized hypotrichosis (Dog 3) and alopecia of the ipsilateral palmar metacarpus (Dog 7). The median duration of treatment with difluprednate ophthalmic emulsion prior to the onset of dermatologic change was 550 days (453–1160 days).

A diagnostic workup was performed for the affected animals beginning with evaluation by a board-certified dermatologist (ADP). Deep skin scrapings of the affected face and pinna revealed adult *Demodex canis* mites with no juvenile stages in one dog from the right face (Dog 7). The quantity and distribution of mites found was not unexpected in a canine receiving steroid treatment and did not explain the cause of the alopecia in areas where *Demodex spp.* mites were not found.

Three-mm skin punch biopsies were collected from the affected areas of the face and pinna of two dogs (Dogs 6 and 7) and submitted for routine histopathologic evaluation by a board-certified veterinary pathologist (EN). The skin biopsies revealed severe follicular atrophy, mild/moderate follicular keratosis, and mild/moderate orthokeratosis (Figs. 1 and 2). These findings were consistent with non-inflammatory alopecia suspected to be secondary to corticosteroid use. Dog 7 also had intrafollicular *Demodex spp.* mites in the biopsy specimen from the right face, consistent with the deep skin scraping results (Fig. 2). However, in the face of the concurrent follicular atrophy, and the few number of mites present without associated inflammation, they were not suspected to be the underlying cause of the alopecia. Following the results of skin scrapings and skin biopsies both Dogs 6 and 7 were reduced from treatment with difluprednate 0.05% ophthalmic emulsion from both eyes (OU) (AAV-treated and AAV-untreated fellow control eye) twice daily (BID) to a single eye (AAV-treated eye only) BID. Hair regrew on the untreated side of the face, while regrowth on the pinna was variable (Figs. 1 and 2).

The high suspicion of adverse effects secondary to ophthalmic corticosteroid use prompted additional diagnostic testing including a complete blood count (CBC) and serum biochemistry (Dogs 6, 7, and 8), ACTH stimulation testing (13/13 dogs). The lymphocyte count, neutrophil count, alkaline phosphatase activity, platelet count, and cholesterol were all within normal limits. Endogenous ACTH plasma concentrations were also measured to further assess the HPA axis.

The results of the ACTH stimulation testing showed that in 8/9 dogs treated with topical difluprednate endogenous ACTH levels were below the reference interval (Table 2). Additionally, baseline cortisol concentrations were below the reference interval in 6/9 dogs, and post-ACTH stimulation cortisol concentrations were below the reference interval in 6/9 dogs and near the lower end of the reference interval in 2/9 dogs (Table 2). A single non-steroid treated control dog (Dog 12) had a mildly decreased endogenous ACTH concentration, while all control dogs (4/4) had normal baseline and post-ACTH stimulation cortisol concentrations.

**Table 2:** Endogenous plasma ACTH and baseline/post-stimulation serum cortisol concentrations

Dog	Endogenous ACTH Range: 6.7–25.0 pmol/L	Baseline Cortisol Range: 15–110 nmol/L	Post-Stimulation Cortisol Range: 220–550 nmol/L
1a	**5.8**	**< 5.5**	259
1b	**5.8**	36	259
2	**5.1**	**< 5.5**	**48**
3a	**3.7**	**< 5.5**	**< 5.5**
3b	**4.8**	**< 5.5**	**< 5.5**
4	**4.9**	**< 5.5**	**< 5.5**
5	**5.6**	25	386
6	15.3	52	**185**
7	**4.7**	29	284
8a	**3.9**	**< 5.5**	**< 5.5**
8b	**3.9**	**< 5.5**	**< 5.5**
9	**3.1**	**< 5.5**	**< 5.5**
10	6.8	65	372
11	**4.7**	72	356
12	14.2	45	227
13	6.9	55	290
14	**3.3**	**< 5.5**	**< 5.5**

Dogs with repeated ACTH stimulation testing annotated as “a” and “b” to reflect chronologic order of testing. Abnormal results are bolded

Dogs that received higher frequency of treatment, or treatment OU, appeared to have more pronounced suppression of the HPA axis. All dogs that were receiving difluprednate OU TID (Dogs 3, 8 and 9) exhibited complete suppression of the HPA axis and did not have quantifiable serum cortisol concentrations prior to, or following, administration of synthetic ACTH. All three dogs also had suppression of endogenous ACTH concentrations. Dogs that received difluprednate in only one eye, or at a more infrequent schedule, demonstrated a more variable degree of suppression of the HPA axis (Table 2).

Based on concern for iatrogenic hyperadrenocorticism, the decision was made to reduce treatment with difluprednate 0.05% ophthalmic emulsion from OU (AAV-treated and AAV-untreated fellow control eye) to a single eye (AAV-treated eye only) treatment in two dogs (Dogs 1 and 3). ACTH stimulation testing was repeated in these dogs approximately 3 months later and demonstrated mild improvements in the degree of HPA axis suppression in Dog 1 (Table 2). ACTH stimulation testing was also repeated in Dog 8 three months after initial testing in which no changes were made to the ophthalmic corticosteroid protocol. Endogenous ACTH, baseline cortisol, and post-ACTH stimulation cortisol concentrations remained low (Table 2). A CBC and serum biochemistry were also performed which were normal.

Approximately 5 months following reduction of the difluprednate dose, an additional skin biopsy was performed in Dog 7 in the area of hair regrowth on the face under heavy sedation. A 3-mm punch biopsy was collected from the skin of the right face and was consistent with mild follicular atrophy (Fig. 2).

### Client owned Chihuahua

A 7-year old female spayed Chihuahua weighing 5.9 kg (Dog 14) was presented to the Michigan State University Veterinary Medical Center (MSU-VMC) Comparative Ophthalmology Service with clinical signs concerning for iatrogenic hyperadrenocorticism following long-term treatment with difluprednate 0.05% ophthalmic emulsion. Written informed consent was obtained from the owner for inclusion in this study. Prior to presentation the dog was diagnosed with anterior uveitis by a board-certified veterinary ophthalmologist. The patient was documented to have elevated serum titers for *Ehrlichia canis, Rickettsia rickettsii, Anaplasma phagocytophilum, and Borrelia burgdorferi*. She was initially started on medical therapy including ketorolac 0.5% ophthalmic solution (OU q6–12 h.; Alcon Laboratories Inc.; Fort Worth, TX), prednisolone acetate 1% ophthalmic suspension (OU q6–12 h.; Alcon Laboratories Inc.; Fort Worth, TX), atropine 1% ophthalmic solution (OU q24 hr.; Akorn Inc.; Lake Forest, IL), and doxycycline hyclate 25 mg tablet (4.25 mg/kg PO q12 hr.).

Three weeks following initiation of treatment, a course of tapering oral prednisolone (0.45 mg/kg PO q12 hr.) was prescribed by the original ophthalmologist because of concerns regarding posterior uveitis. In addition, mycophenolate mofetil oral suspension was also prescribed (10 mg/kg PO q12 hr.) for 5 days, followed by a dose-reduction for long-term treatment (10 mg/kg PO q24 hr.). The panuveitis persisted despite aggressive therapy and was suspected to be immune-mediated in origin.

**Fig. 3:**
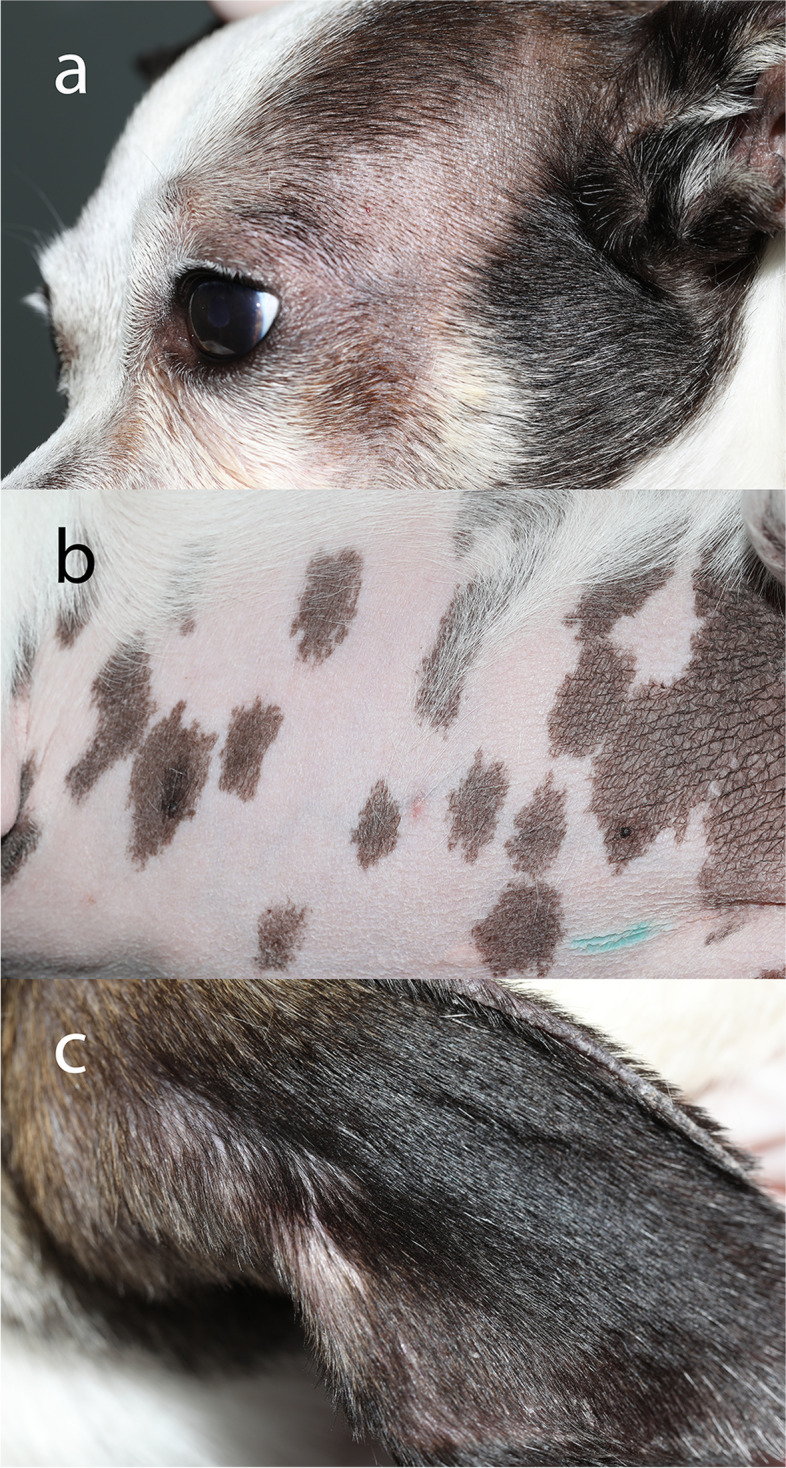
Clinical photographs from Dog 14 following approximately 13 months of treatment with difluprednate 0.05% ophthalmic emulsion OU BID-TID. Diffuse hypotrichosis of the head (**a**), alopecia and thinning of skin on the ventral abdomen (**b**), and mild hypotrichosis of the right ear (**c**)

Approximately 18 months into therapy, the patient was switched from prednisolone acetate 1% ophthalmic suspension to difluprednate 0.05% ophthalmic emulsion by the MSU-VMC Comparative Ophthalmology Service. She was initially started at 1 drop OU q8 hr. and tapered to 1 drop OU q12 hr. 6 months into treatment. Control of her anterior uveitis improved with treatment. The dog was presented to the primary care veterinarian for concerns of a potbellied appearance, hair loss, polyuria, and polydipsia 413 days following initiation of difluprednate 0.05% ophthalmic emulsion (Fig. 3).

Because of a high index of suspicion for iatrogenic hyperadrenocorticism, a CBC, serum chemistry, urinalysis, and ACTH stimulation test were submitted by the primary care veterinarian. Results revealed a mildly increased ALP activity of 194 U/L (5–160 U/L), no abnormalities on the CBC, and a urine specific gravity of 1.016. Urine sediment evaluation also revealed bacteriuria. Total T4 was evaluated as a part of the comprehensive panel and was within the reference interval. The ACTH stimulation test revealed marked suppression of endogenous ACTH, baseline cortisol, and post-ACTH stimulation cortisol concentrations (Table 2).

Based on the laboratory results, the difluprednate 0.05% ophthalmic emulsion was tapered to 1 drop OU q24 hr., and her polyuria and polydipsia improved. Over the following 3 weeks the alopecia began to improve on the head and extremities; however, thinning of the skin and alopecia persisted on the ventral abdomen. Attempts were made to reduce her dosing frequency further to 1 drop OU q48 hr., which resulted in worsening of her anterior uveitis. The decision was made to maintain her on 1 drop OU q24 hr., while concurrently increasing the dose of mycophenolate mofetil to 10 mg/kg PO q12 hr. These changes were sufficient to control her uveitis while minimizing her systemic clinical signs. Repeated endocrinology testing was not pursued.

**Fig. 4:**
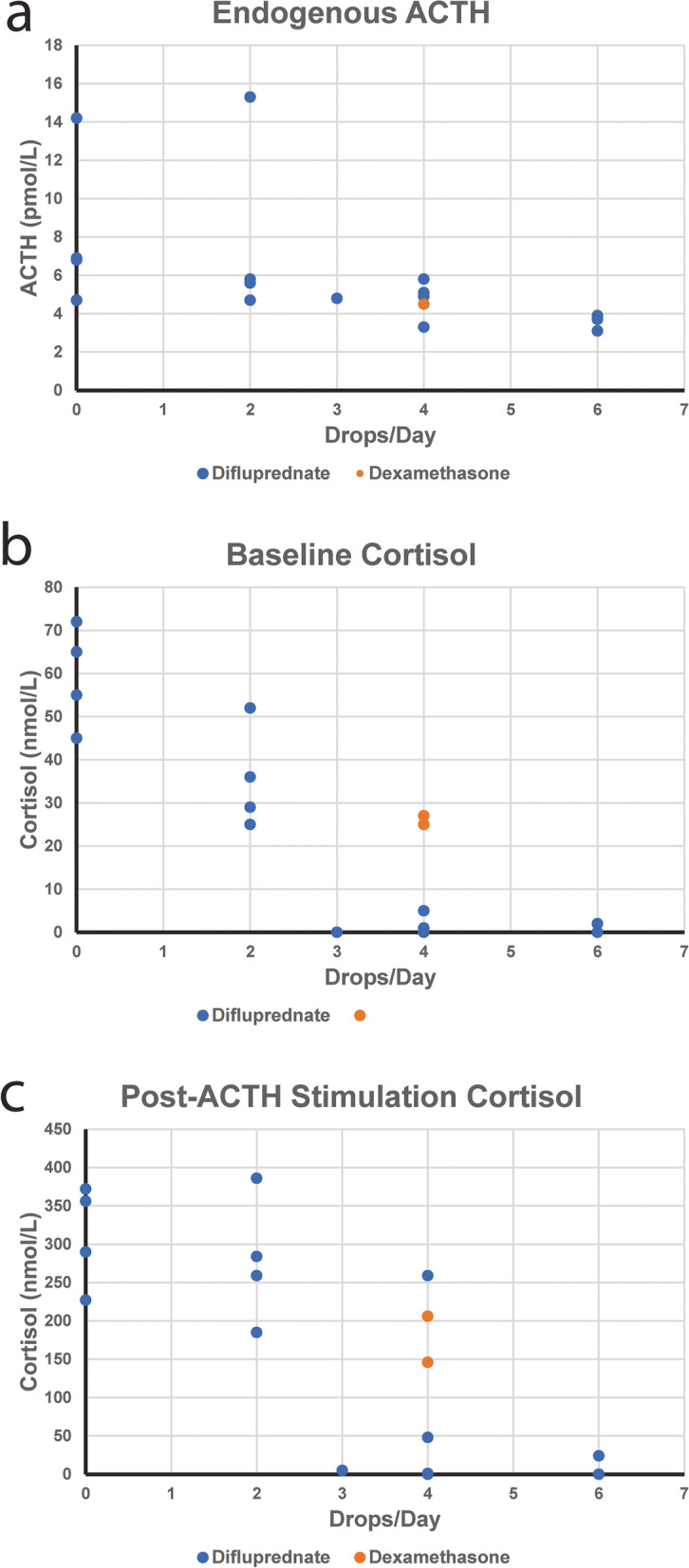
Effect of ophthalmic corticosteroids on HPA axis. Endogenous ACTH (**a**), baseline cortisol (**b**), and post-ACTH stimulation cortisol (**c**) were plotted against the total dose of difluprednate 0.05% ophthalmic emulsion (drops/day). A statistically significant negative correlation was found between the dose of difluprednate 0.05% ophthalmic emulsion and the endogenous ACTH (r = − 0.535, *p* = 0.027) (**a**), baseline cortisol (r = − 0.890; *p* < 0.001) and post-ACTH stimulation cortisol concentrations (r = − 0.817, p < 0.001) (**b,c**)

Pearson’s correlation coefficients were calculated comparing the dose of difluprednate (defined as drops administered per day) to the results of the ACTH stimulation testing for the Beagles and the Chihuahua. The daily dose of difluprednate had a significant negative correlation with the endogenous ACTH (r = − 0.535, *p* = 0.027) as well as both baseline (r = − 0.890, *p* < 0.001) and post-stimulation cortisol (r = − 0.817, p < 0.001) concentrations (Fig. 4).

## Discussion

This report describes the clinical, dermatologic, and laboratory abnormalities within a colony of research Beagles and an unrelated clinical patient following long-term treatment with difluprednate 0.05% ophthalmic emulsion. Focal endocrine alopecia and suppression of the HPA axis as reported herein have not been reported previously in dogs following treatment with difluprednate ophthalmic emulsion. Difluprednate is a potent fluorinated corticosteroid which has superior anti-inflammatory effects compared to other commercially prepared ophthalmic corticosteroids [11]. The combined improved potency and penetration results in an increased risk for fluorinated steroids to cause unwanted local and systemic adverse effects [20].

The first clinical sign in our treated research Beagles was alopecia affecting the periocular area and pinna. Even though there was evidence of systemic absorption of corticosteroids based upon the suppression of the HPA axis in all treated dogs, the periocular and pinna alopecia in the Beagles were felt to be secondary to local effects from dermal absorption of the difluprednate. We believe that Dog 14 (Chihuahua) demonstrated changes more consistent with iatrogenic hyperadrenocorticism.

The dogs treated with ophthalmic corticosteroids showed variable suppression of the HPA axis with every dog having at least one tested parameter below the established reference interval. We demonstrated that there were significant negative correlations between the dose of difluprednate and the results of the ACTH stimulation testing (endogenous ACTH, baseline cortisol, and post-stimulation cortisol concentrations). This is supportive of systemic absorption and subsequent dose-dependent negative feedback on the HPA axis. We did not find that this trend was mirrored by presence or severity of dermatologic changes. The dogs with the most severe suppression on ACTH stimulation testing had no appreciable dermatologic changes, and dogs with marked alopecic changes had marginally abnormal testing.

While the duration of corticosteroid treatment varied, long-term therapy appeared consistent amongst all affected dogs in this study. The laboratory Beagles were treated for a median of 550 days (453–1160 days) prior to detection of dermatologic changes. The duration of treatment with difluprednate and variation in individual sensitivity to corticosteroids may have resulted in more diffuse dermatologic changes appreciated in Dog 3 compared to the other laboratory Beagles.

Increased circulating corticosteroids cause predictable changes to the CBC and serum biochemistry [1]. Despite all dogs exhibiting some degree of suppression of the HPA axis, and some dogs developing significant dermatologic changes, the clinical case (Dog 14) was the only dog in this case series to develop biochemical abnormalities– a mildly increased serum ALP activity. We suspect that the level of systemic absorption may not have been at a level high enough to induce changes to the CBC and biochemistry in the Beagles. This may also be reflective of individual variability in sensitivity to exogenous corticosteroids.

The most consistent dermatologic change appreciated in the laboratory Beagles was alopecia primarily localized to the head; however, Dog 3 also exhibited generalized mild hypotrichosis. This dog also had significant suppression of the HPA axis and had been treated with difluprednate for the longest duration prior to the onset of dermatologic changes (1160 days). The typical difluprednate-induced abnormalities described here contrast dermatologic changes observed following long-term use of ophthalmic NPD in a laboratory Beagle not included in this study: This dog developed bilateral, symmetrical flank alopecia, but did not have any hair loss on the face or ears (Additional file 1).

Our observations in the laboratory Beagles are contrasted with the Chihuahua (Dog 14), which exhibited many classic physical examination changes, clinical signs (polyuria and polydipsia) in addition to the laboratory changes associated with hyperadrenocorticism. This dog had also received long-term therapy with difluprednate prior to diagnosis with iatrogenic hyperadrenocorticism (413 days). We suspect that the overall dose of difluprednate contributed to the difference in presentations between the Beagle dogs and the clinical case. The median weight of the Beagle dogs was over two times that of the Chihuahua, and while they received very similar dosing protocols, the Chihuahua had a larger overall dose when adjusting for the difference in body weight.

The Chihuahua (Dog 14) had generalized hypotrichosis of the head and body without distinct localization to the periocular area or pinna. These clinical signs were alleviated following reduction in her difluprednate dose. Ideally this dog would have been tapered off difluprednate to correct the suppression of the HPA axis; however, her disease could not be controlled with less frequent treatment and other ophthalmic anti-inflammatory medications. The decision was made to keep her on the lowest effective dose of difluprednate (1 drop OU q24hr.) to minimize clinical signs associated with steroid use, while still controlling her uveitis, and minimizing the risk of vision loss or secondary glaucoma.

Hair was observed to regrow on the non-treated side of the face once administration was reduced to unilateral therapy in the difluprednate treated Beagles. This supports our hypothesis that difluprednate was acting locally on the tissue, and most likely contacted the face through overflow of the tear film around the time of medicating. The pinna also likely came into direct contact with difluprednate during head shaking behavior, as Beagles have long heavy ears that are mobile during head shaking. Dog 7 was observed to have alopecia of the palmar metacarpus ipsilateral to the treated eye. The limb likely contacted difluprednate through face rubbing or grooming behaviors. Dog 6 did not regrow the hair of the distal ipsilateral pinna AS following discontinuing difluprednate OS. We suspect that continued corticosteroid use OD resulted in sufficient systemic absorption to delay hair regrowth AS.

Several limitations exist with this study. The first is that some data was collected in a retrospective manner including the identification of the onset of clinical signs (alopecia) within both the Beagles and the Chihuahua. Additionally, not every diagnostic test was performed on all dogs. While all dogs had ACTH stimulation testing performed, only a limited number of dogs had skin biopsy, CBC, and serum biochemistry performed. While efforts were made to test representative dogs within the research colony, dogs with biochemical abnormalities may have been missed. This study is also limited by the number of cases, which precludes from statistical analysis to draw significance regarding steroid dose and duration of treatment prior to the onset of clinical signs. Finally, the housing of the laboratory Beagles does not allow us to comment on the clinical signs that are frequently cited with hyperadrenocorticism, such as polyuria and polydipsia. These were not documented in the medical records of our group-housed research Beagles because drinking and urination habits were not quantified.

## Conclusions

In conclusion, this study describes local and systemic adverse effects and suppression of the HPA axis in dogs following long-term treatment with difluprednate 0.05% ophthalmic emulsion. To our knowledge this is the first report of localized alopecia to the face, periocular region, and pinna following treatment with topical ophthalmic corticosteroids in dogs. We hypothesize that the high potency and penetration of difluprednate is a contributing and possible causative factor to the localized alopecia. An increased risk for suppression of the HPA axis and iatrogenic hyperadrenocorticism should also be considered when using difluprednate 0.05% ophthalmic emulsion.

## Methods

### Dogs

Among the 13 purpose-bred adult Beagles were 5 intact males, 1 neutered male, and 7 intact females (Table 1). The median age was 3 years (2–5 years). The median weight of the dogs was 12.6 kg (9.6–17.6 kg). Genotypes were confirmed based on *ADAMTS10* gene sequence: glaucomatous dogs were homozygous for the G661R missense mutation which is responsible for OAG in Beagles, while the normal dogs were either carriers of the mutation or homozygous for the wild-type allele [19]. All laboratory Beagles belonged to the principal investigator (AMK) and were group-housed in the same facility at Michigan State University College of Veterinary Medicine with a 12-h/12-h light/dark cycle and fed the same diet (Teklad 2027; Envigo; Madison, WI). Except for two dogs (Dogs 1 and 6), all animals were born and raised in our research facility. Dogs 1 and 5 were purchased from a commercial vendor (Marshall BioResources; North Rose, NY). At the conclusion of this study the dogs remained in the research colony and enrolled in other concurrent studies. No dogs were euthanized for the purposes of this study.

The 7-year old female spayed Chihuahua was privately-owned and presented to MSU-VMC Comparative Ophthalmology Service as a clinical patient.

The studies were done in accordance with the Association of Research in Vision and Ophthalmology (ARVO) Statement for the Use of Animals in Ophthalmic Vision and Research and approved by the Michigan State University Institutional Animal Care and Use Committee (IACUC).

### ACTH stimulation test and endogenous ACTH plasma concentrations

The ACTH stimulation test was performed by measuring serum cortisol concentrations immediately before and 1 h after intravenous administration of 5 μg/kg synthetic ACTH (Cosyntropin, Oakwood Laboratories LLC, Solon, OH, for Sandoz Inc., Princeton, NJ). Samples for cortisol and ACTH were evaluated at the Michigan State University Veterinary Diagnostic Laboratory, which is an American Association of Veterinary Laboratory Diagnosticians accredited laboratory. Serum cortisol concentrations and plasma ACTH concentrations were determined using a commercially available competitive chemiluminescent immunoassay (Immulite® 2000 Cortisol, Siemens Healthcare Diagnostics Ltd., Gwynedd, UK) and an immunoradiometric assay (ACTH Immunoradiometric Assay, Scantibodies Laboratory, Inc., Santee, CA), respectively [21, 22].

### Histopathology

All collected formalin fixed tissue was submitted for routine histopathologic evaluation by the same board-certified veterinary pathologist (EN). A BX43F Microscope (Olympus, Waltham, MA), UC90 9MP Color Camera, and cellSens Standard Software (Olympus, Waltham, MA) were used to obtain microscopy images. Images were obtained at 96 pixels per inch, and then processed to a final resolution of 400 pixels per inch using Adobe Photoshop (Adobe, Inc., San Jose, CA) for manuscript figures (Figs. 1 and 2, Additional file 1).

### Statistics

A Pearson’s correlation coefficient was calculated comparing the dose of difluprednate (defined as drops administered per day) to the results of the ACTH stimulation testing for the Beagles and the Chihuahua, and *p*-values ≤0.05 were considered significant for all comparisons (Excel; Microsoft® Corporation, Redmond, WA).

## Supplementary Information

**Additional file 1.** Clinical and histopathologic abnormalities in a laboratory Beagle following long-term use of ophthalmic NPD. Clinical photographs show mild epiphora OS, but no appreciable areas of facial or pinna alopecia following 28 months of treatment with NPD OU BID (a). Marked alopecia along the ventral abdomen with numerous comedones (b). Left flank with appreciable thinning of hair coat (c). Photomicrograph from a skin biopsy of the right flank (H&E) (d). There is mild/moderate follicular atrophy, mild/moderate follicular keratosis, and moderate orthokeratosis (d). Scale bar = 400 μm.

## Data Availability

All data generated or analyzed during this study are included in this published article.

## Abbreviations

AAV: Adeno-associated virus
ACTH: Adrenocorticotropic hormone
ALP: Alkaline phosphatase
ALT: Alanine aminotransferase
ARVO: Association of Research in Vision and Ophthalmology
BID: Twice daily
CBC: Complete blood count
HPA axis: Hypothalamic-pituitary-adrenal axis
IACUC: Institutional Animal Care and Use Committee
MSU-VMC: Michigan State University, Veterinary Medical Center
NPD: Neomycin-polymyxin B-dexamethasone
OAG: Open-angle glaucoma
OD: Oculus Dexter, right eye
OS: Oculus Sinister, left eye
OU: Oculus Uterque, both eyes
T3: Total triiodothyronine
T4: Thyroxine
TID: Three times daily
TSH: Thyroid stimulating hormone
TT4: Total thyroxine
