# Supplementary figures and images for: Localized alopecia and suppression of hypothalamic-pituitary-adrenal (HPA) axis in dogs following treatment with difluprednate 0.05% ophthalmic emulsion (Durezol®)
Source: BMC Vet Res. 2021 Dec 1;17:366. doi: 10.1186/s12917-021-03072-9 (PMC8638448; doi:10.1186/s12917-021-03072-9)

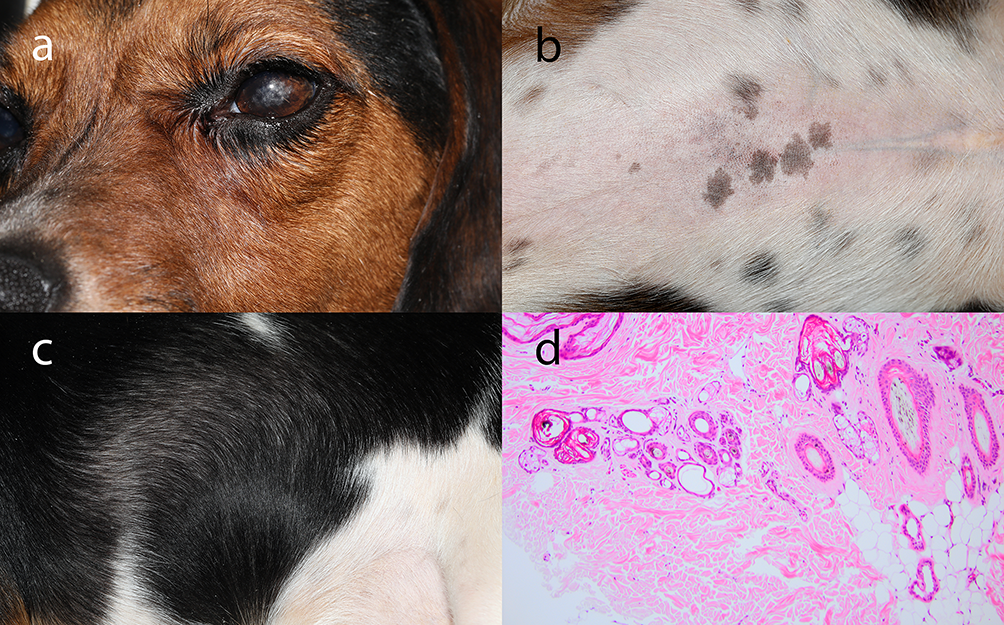

Supplement: Supplementary file 1 — Additional file 1. Clinical and histopathologic abnormalities in a laboratory Beagle following long-term use of ophthalmic NPD. Clinical photographs show mild epiphora OS, but no appreciable areas of facial or pinna alopecia following 28 months of treatment with NPD OU BID (a). Marked alopecia along the ventral abdomen with numerous comedones (b). Left flank with appreciable thinning of hair coat (c). Photomicrograph from a skin biopsy of the right flank (H&E) (d). There is mild/moderate follicular atrophy, mild/moderate follicular keratosis, and moderate orthokeratosis (d). Scale bar = 400 μm. [file 12917_2021_3072_MOESM1_ESM.tif]
